# Deficiency of TLR4 homologue RP105 aggravates outward remodeling in a murine model of arteriovenous fistula failure

**DOI:** 10.1038/s41598-017-10108-4

**Published:** 2017-08-31

**Authors:** Taisiya Bezhaeva, ChunYu Wong, Margreet R. de Vries, Eric P. van der Veer, Carla M. A. van Alem, Ivo Que, Reshma A. Lalai, Anton-Jan van Zonneveld, Joris I. Rotmans, Paul H. A. Quax

**Affiliations:** 1 Department of Internal Medicine, Leiden University Medical Center, Leiden, The Netherlands; 2 Einthoven Laboratory for Experimental Vascular Medicine, Leiden University Medical Center, Leiden, The Netherlands; 3 Department of Vascular Surgery, Leiden University Medical Center, Leiden, The Netherlands; 4 Department of Radiology, Leiden University Medical Center, Leiden, The Netherlands

## Abstract

Arteriovenous access dysfunction is a major cause of morbidity for hemodialysis patients. The pathophysiology of arteriovenous fistula (AVF) maturation failure is associated with inflammation, impaired outward remodeling (OR) and intimal hyperplasia. RP105 is a critical physiologic regulator of TLR4 signaling in numerous cell types. In the present study, we investigated the impact of RP105 on AVF maturation, and defined cell-specific effects of RP105 on macrophages and vascular smooth muscle cells (VSMCs). Overall, RP105^−/−^ mice displayed a 26% decrease in venous OR. The inflammatory response in RP105^−/−^ mice was characterized by accumulation of anti-inflammatory macrophages, a 76% decrease in pro- inflammatory macrophages, a 70% reduction in T-cells and a 50% decrease in MMP-activity. *In vitro*, anti-inflammatory macrophages from RP105^−/−^ mice displayed increased IL10 production, while MCP1 and IL6 levels secreted by pro-inflammatory macrophages were elevated. VSMC content in RP105^−/−^ AVFs was markedly decreased. *In vitro*, RP105^−/−^ venous VSMCs proliferation was 50% lower, whereas arterial VSMCs displayed a 50% decrease in migration, relative to WT. In conclusion, the impaired venous OR in RP105^−/−^ mice could result from of a shift in both macrophages and VSMCs towards a regenerative phenotype, identifying a novel relationship between inflammation and VSMC function in AVF maturation.

## Introduction

The placement of an arteriovenous fistula (AVF) is currently regarded as the best available option for permanent vascular access in patients requiring chronic hemodialysis. For proper maturation of the AVFs, both a major increase in blood flow and venous diameter are required to allow adequate hemodialysis treatment. However, several clinical trials have shown that the 1-year primary patency rate of AVFs does not exceed 60%, illustrating the fact that the need for further improvement of this access conduit is vital^1, 2^. AVF-related complications are frequently encountered shortly after AVF surgery, as 30–60% of the AVF fail to mature adequately to support dialysis therapy^3^. The exact mechanisms that lead to AVF maturation failure remain unknown, but both impaired outward remodeling (OR) and formation of intimal hyperplasia (IH) are regarded as primary contributors to this pathophysiology^4^. Recent studies have shown that the process of vascular adaptation after AVF creation is associated with an excessive inflammatory response^5–8^ and proliferation and migration of arterial and venous vascular smooth muscle cells (VSMCs) towards the intima at the site of anastomosis^9–11^. In view of extensive adverse consequences resulting from AVF failure and the subsequent burden for hemodialysis patients, there is increasing emphasis on pathophysiological studies aimed to unravel the complex mechanisms underlying AVF failure. The latter is pivotal in efforts to identify novel molecular therapeutic targets that could potentially improve AVF patency.

Toll-like receptor 4 (TLR4) is a well-known sentry that induces a pro-inflammatory signaling cascade^12^. Its function is modulated not only by exogenous pathogens in the context of microbial infections^13^, but also by several endogenous stimuli in inflammatory conditions such as atherosclerosis^14, 15^ or during vascular remodeling^16–19^. To initiate the TLR4 signaling cascade, activation of its adaptor molecule MD2 is required which is responsible for the recognition of bacterial lipopolysaccharide (LPS) on the cell surface^20^. Due to the hierarchical importance of TLR4 in the innate immune response and its ubiquitous function, the signaling activity of this protein is firmly regulated by several regulatory molecules. One such regulator is RP105 (radioprotective 105, also known as CD180), a cell surface protein expressed by numerous cell types, including inflammatory cells and VSMCs^21, 22^. The structure of RP105 is evolutionarily similar to TLR4 and it associates with MD1, a MD2 homologue which promotes RP105 cell surface expression^23, 24^. RP105–MD1 exerts dichotomous regulatory activities on TLR4-mediated LPS responses in a cell type-dependent fashion^25^. On B-cells, RP105-MD1 drives cellular proliferation and enhances B-cell- dependent inflammatory processes^26^. In contrast, in myeloid cells, RP105 acts as a natural antagonist of TLR4 signaling^27^, while the functional role in VSMCs remains poorly understood. Previous studies from our group have demonstrated that RP105 deficiency results in decreased atherosclerotic lesion formation via alterations on pro-inflammatory B-cells^28^ and a CCR2-dependent decrease in monocyte influx^29^. Strikingly, complete opposite effects were observed in a murine model of vein graft disease, where a 90% increase in graft lesion area was linked to a local increase in macrophage content and lesional levels of monocyte chemoattractant protein-1 (MCP1), expressed by VSMCs^21^. In a model of post-interventional vascular remodeling, artery cuff placement in RP105^−/−^ mice resulted in increased neointima formation, which coincided with an increase in arterial VSMC proliferation *ex vivo* ^22^.

In the context of both AVF maturation and failure, numerous cell types are involved including inflammatory cells and VSMCs from both the feeding artery^10, 30^ and local venous wall^31^, making it a unique model to unravel specific functional consequences of RP105 on remodeling in AVF.

In the present study, we aimed to elucidate the role of RP105 on AVF maturation in a murine model of AVF failure by assessing cell type-specific effects of RP105 deficiency, on macrophage polarization and VSMC behavior.

## Results

### RP105 deficiency influence AVF maturation

**Figure 1:**
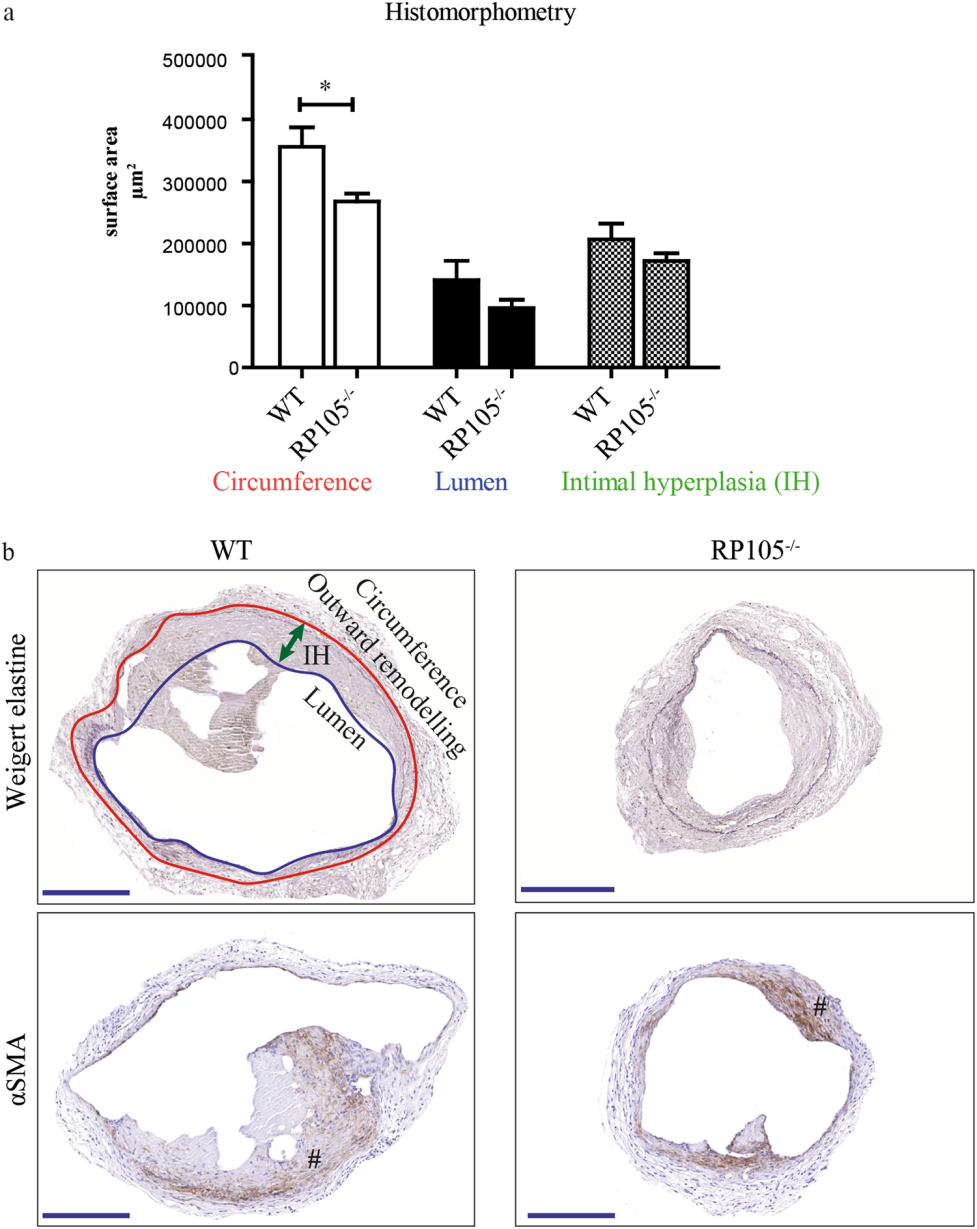
Effect of RP105 deficiency on AVF maturation *in vivo*. (**a**) Quantification of morphometric parameters. Decrease in vessel circumference (outward remodeling) in RP105 deficient mice was observed 14 days after AVF creation, compared to WT. Lumen and intimal hyperplasia did not differ between RP105^−/−^ and WT mice. (**b**) Histological staining of venous outflow tract 14 days after surgery. Weigert elastine staining was used to determine histomorphometrical parameters of the vessel. Circumference (internal elastic lamina area) was used to quantify outward remodeling (red line). Intimal hyperplasia (green arrow) measured as a difference between luminal area (blue line) and vessel circumference. αSMA staining shows area of intimal hyperplasia 14 days after AVF creation. (#) intimal hyperplasia; P < 0.05; n = 11 per group. Bar = 200 μm; 100x magnification.

To investigate how differential expression of RP105 could influence AVF maturation, we created an AVF by an end-to-side ligation of the jugular vein to the carotid artery of wild- type (WT) and RP105^−/−^. Two weeks after surgery the tissue was processed to paraffin, and 5-µm sections were made perpendicular to the vein at 12 locations with an interval of 150 µm. Because most of the stenotic lesions in human AVFs occur in the venous outflow tract we analyzed first 3 consecutive venous sections downstream from the area closest to the anastomosis. AVF material was evaluated using morphometric and immunohistochemical approaches. RP105^−/−^ mice showed a 26% smaller circumference of the external jugular vein compared to WT mice (P = 0.03) (Fig. 1a), indicating that RP105 deficiency impacts outward remodeling. As shown in Fig. 1b, diminished RP105 expression did not influence IH in the venous outflow tract of the AVF. Importantly, immunohistochemical staining revealed that the vast majority of intimal cells are αSMA^+^ in both WT and RP105^−/−^ mice (Fig. 1b).

### RP105 deficiency leads to reduced VSMC proliferation in AVF lesions

**Figure 2:**
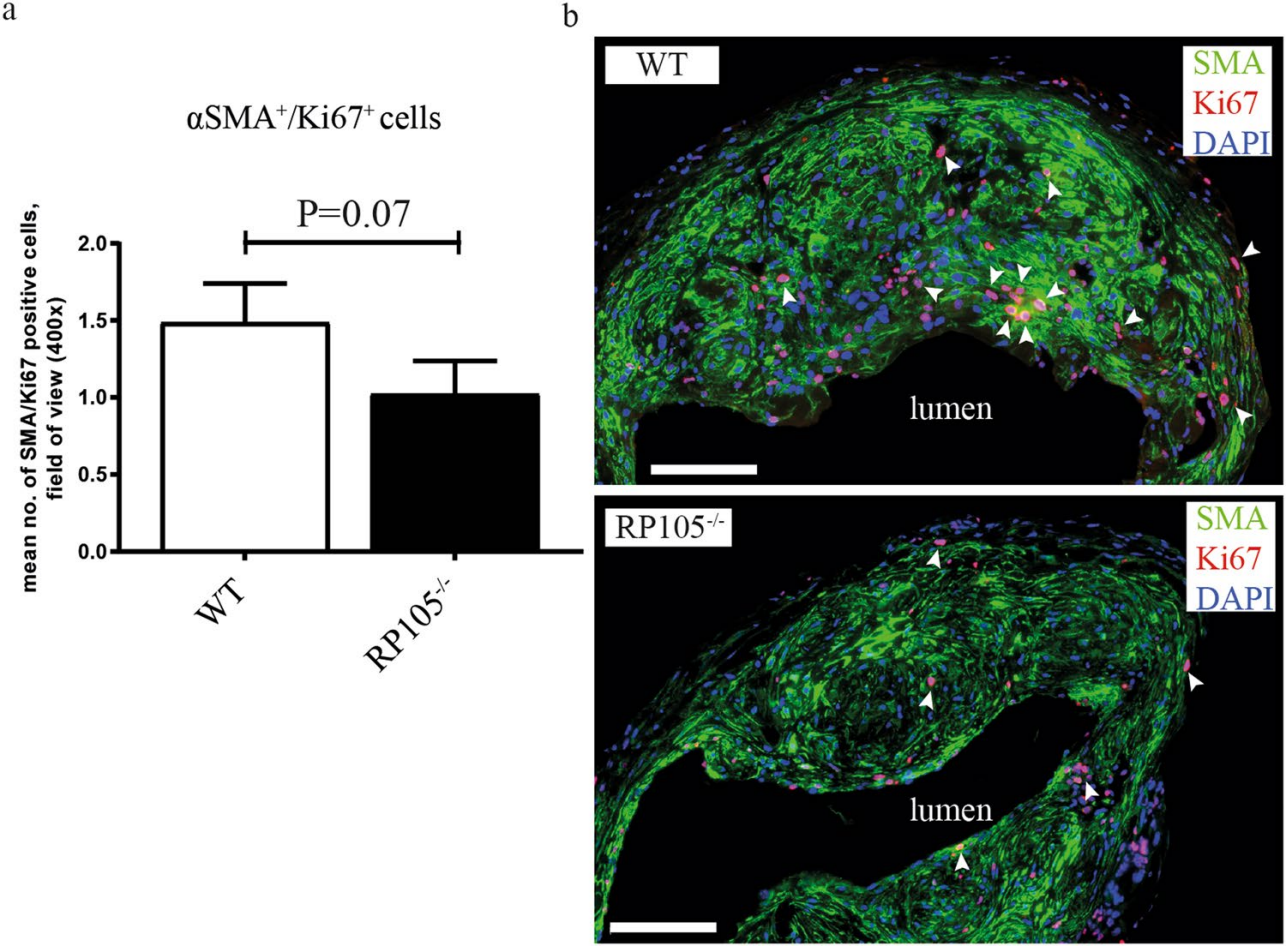
Effect of RP105 deficiency on VSMCs proliferation *in vivo*. Quantification (**a**) and immunofluorescent staining (**b**) of αSMA^+^/Ki67^+^ cells (white arrows) revealed reduction in number of proliferating VSMCs in AVF lesions of RP105^−/−^ mice compared to WT 14 days after AVF surgery. n = 11 per group. Bar = 100 μm.

Given this VSMC enrichment in the intimal region of mature AVF, we sought to determine the proliferation capacity of these cells immunohistochemically. For this, we quantitated the amount of proliferating αSMA^+^/Ki67^+^ cells in AVF sections. These studies revealed a 31% decrease in αSMA^+^/Ki67^+^ VSMCs in RP105^−/−^ mice, as compared to WT mice (Fig. 2a).

Since both arterial and venous VSMC might contribute to the portion of proliferating VSMC and we cannot discriminate between arterial and venous VSMCs *in vivo*, this borderline significance in the number of αSMA^+^/Ki67^+^ VSMCs (P = 0.07) might be relevant (Fig. 2b).

### Diminution of RP105 differently affects arterial and venous VSMC function

**Figure 3:**
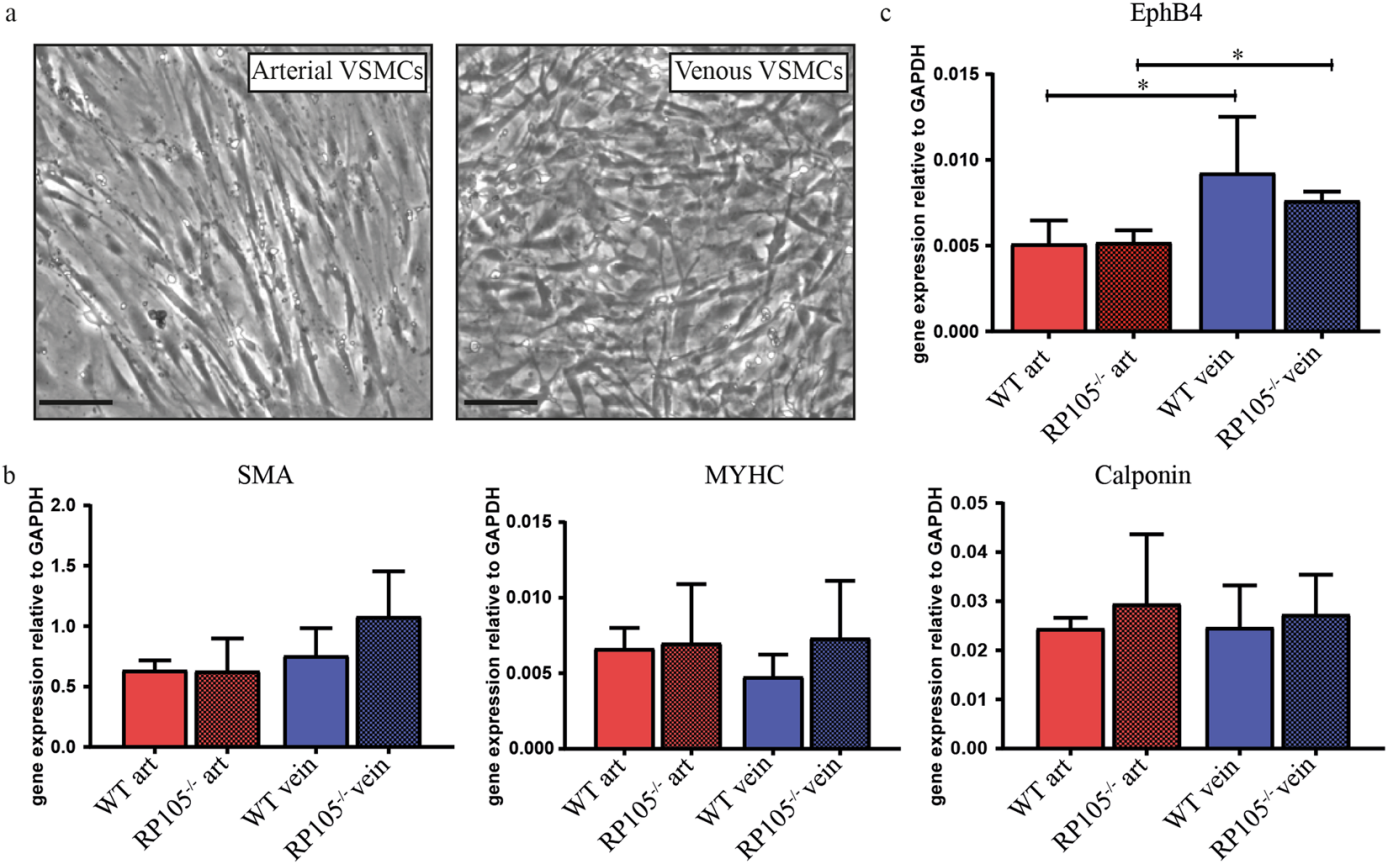
*In vitro* cultured arterial and venous SMCs. (**a**) Microscopic representation of morphological difference between cultured arterial and venous VSMCs. Bar = 100 μm; 200x magnification. (**b**) VSMCs phenotype after 14 days in culture was confirmed by the stable mRNA expression levels of smooth muscle α-actin (SMA), myosin heavy chain (MHC) and calponin. (**c**) Stable increase in EphB4 mRNA levels was decreased in venous VSMCs isolated from WT and RP105^−/−^ mice. ^*^P < 0.05; n = 3.

To further dissect the contribution of arterial and venous VSMCs to AVF maturation and failure, we elected to study the consequences of differential RP105 expression in arterial and venous VSMCs *ex vivo*. For this, we cultured explant material from the carotid artery and vena cava of WT and RP105^−/−^ mice for 2 weeks. Morphologically, we observed that arterial VSMCs possessed an elongated phenotype, whereas venous cells had a more stellate appearance (Fig. 3a). Both arterial and venous VSMCs displayed characteristics of differentiated VSMCs, as confirmed by stable gene expression of VSMCs markers (smooth muscle α-actin (SMA), myosin heavy chain (MYHC) and calponin) after 2 weeks of culture (Fig. 3b). The phenotypic difference and the vascular origin of arterial versus venous VSMCs was confirmed by assessing EphB4 expression levels, an established embryological marker of venous origin^32, 33^, which was increased in cultured venous VSMCs up to 2 weeks after isolation (Fig. 3c).

**Figure 4:**
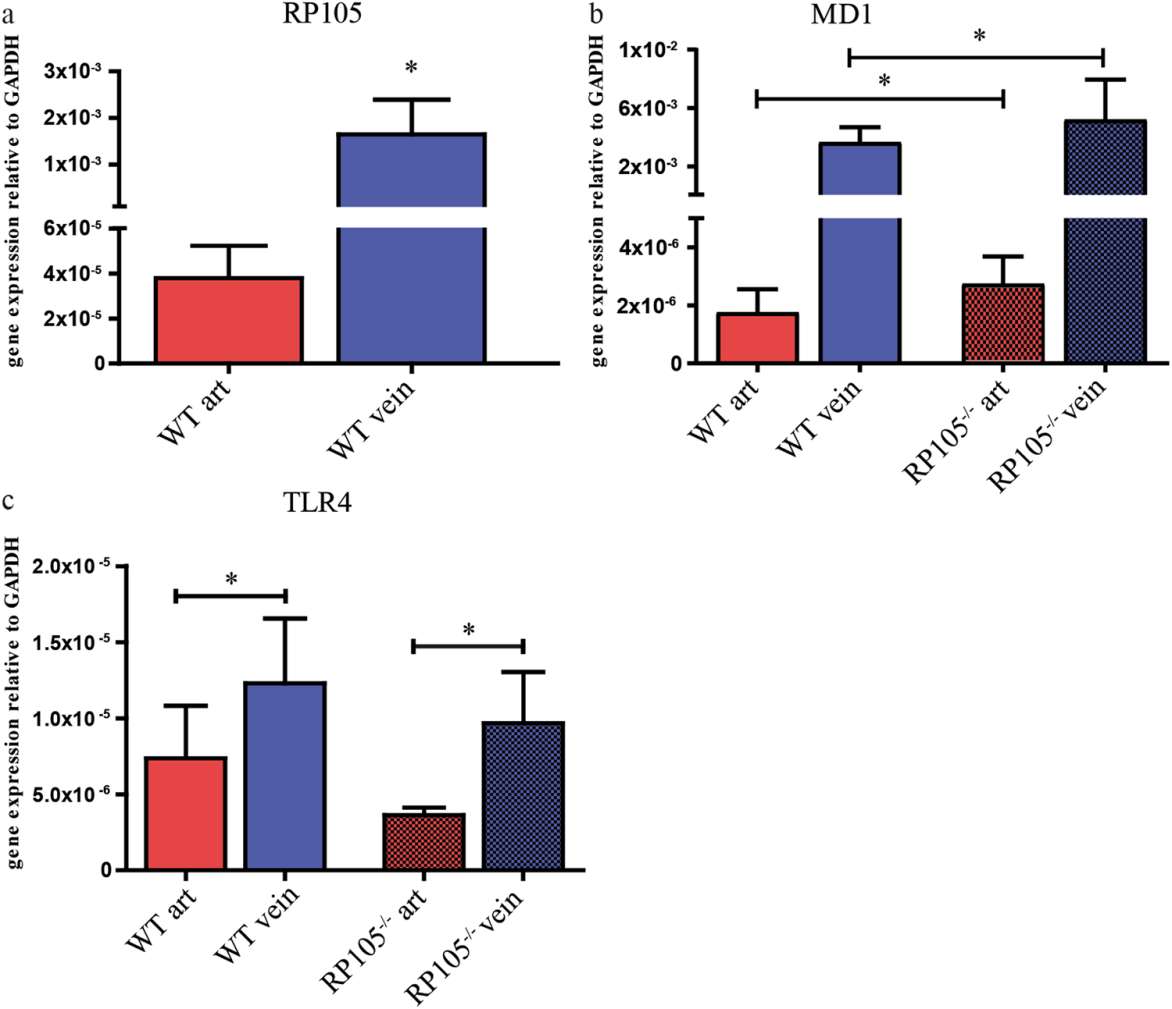
Difference in mRNA expression levels between arterial and venous VSMCs *in vitro*. (**a**) RP105, (**b**) MD1, (**c**) TLR4. The relative expression normalized to GAPDH. ^*^P < 0.05; n = 3.

Next, we further determine expression levels of RP105 associating molecules. Interestingly, RT-PCR analysis of RP105 by WT VSMCs revealed a striking >100-fold increase in gene expression on venous VSMCs compared to arterial VSMCs (Fig. 4a). Expression of its accessory molecule MD1 was also elevated >100-fold on venous VSMCs isolated from both WT and RP105^−/−^ mice (Fig. 4b). mRNA levels of inflammatory marker TLR4 was elevated by 48% in venous cells, as compared to arterial VSMCs (Fig. 4c). Expression of the TLR4 accessory molecule MD2 did not differ between WT and RP105^−/−^ mice arterial and venous VSMCs (Supplementary Figure 1).

**Figure 5:**
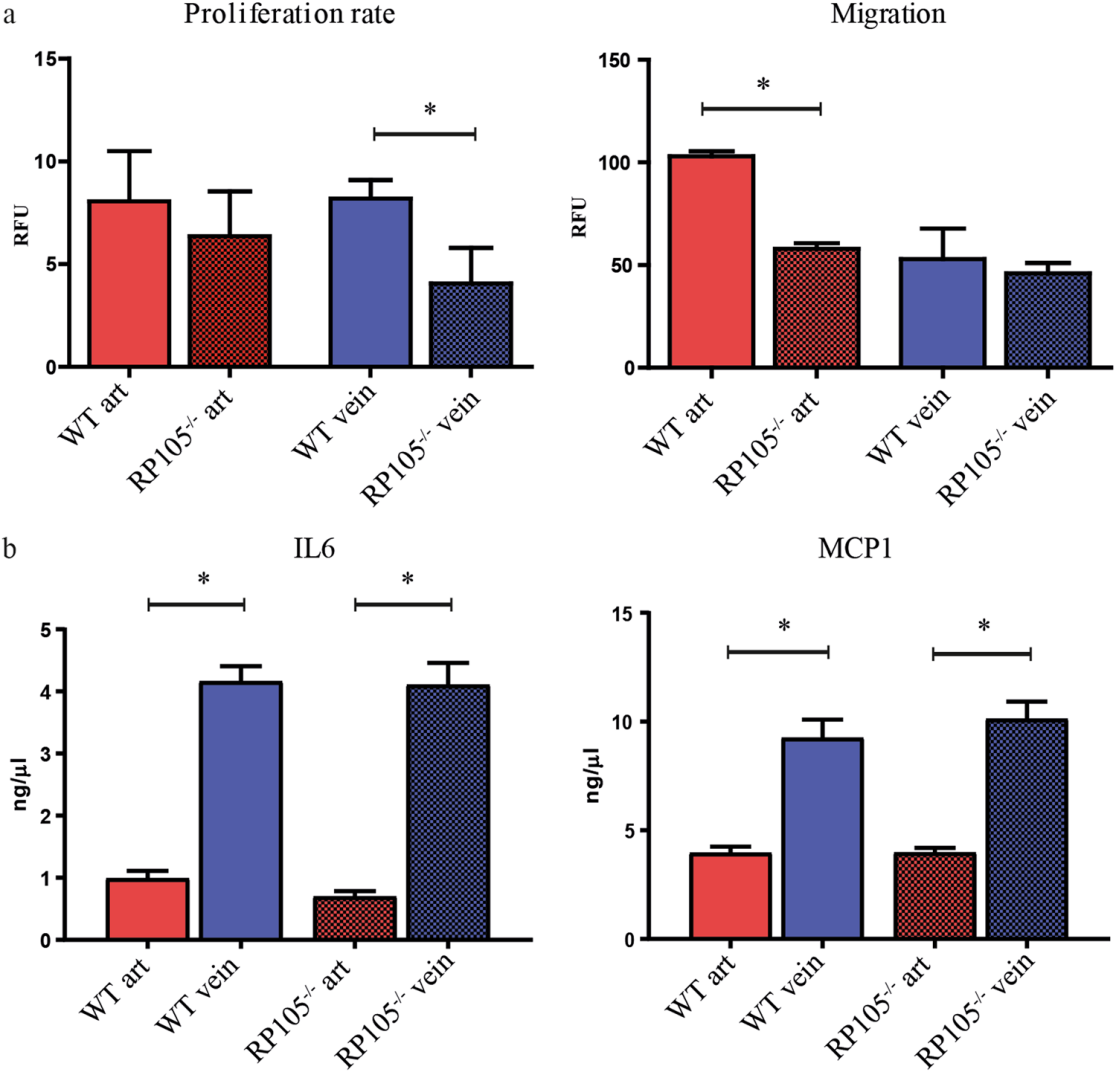
Functional difference between arterial and venous VSMCs *in vitro*. Reduction in proliferation rate was limited to VSMCs isolated from RP105^−/−^ veins. Decrease in migration of VSMCs isolated from RP105^−/−^ mice was restricted to arterial cells only. Proliferation rate and migration were measured over 16 h time period. (**b**) Venous VSMCs isolated from WT and RP105^−/−^ mice produce significantly higher amounts of inflammatory cytokines IL6 and MCP1. Cells were maintain in culture for 14 days. ^*^P < 0.05; n = 3.

Functionally, venous VSMCs derived from RP105^−/−^ mice displayed a 50% reduction in their rate of proliferation, relative to VSMCs derived from WT mice, while arterial VSMCs proliferation was unaltered (Fig. 5a). In contrast, migratory capacity was reduced by 50% over a 16 h time period in arterial VSMCs derived from RP105^−/−^, venous SMCs showed no difference in migration between WT and RP105^−/−^ mice (Fig. 5a). As VSMCs are also potent cytokine producers we measured amount of pro-inflammatory cytokines IL6 and MCP1 secreted by arterial and venous VSMCs from WT and RP105^−/−^ mice. Although there was no difference in IL6 and MCP1 levels between WT and RP105^−/−^, venous VSMCs isolated from both WT and RP105^−/−^ mice exhibited higher inflammatory state characterized by 70% and 84% increase in IL6 secretion by WT and RP105^−/−^ VSMCs respectively when compared to arterial VSMC and 57% and 61% up regulation in MCP1 levels produced by WT and RP105^−/−^ VSMCs respectively when compared to arterial VSMCs (Fig. 5b). There was no difference in the amount of anti-inflammatory cytokine IL10 produced either by arterial or venous VSCMs from WT and RP105^−/−^ VSMCs (Supplementary Figure 2).

### RP105 deficiency impacts the inflammatory status of AVF infiltrating cells

**Figure 6:**
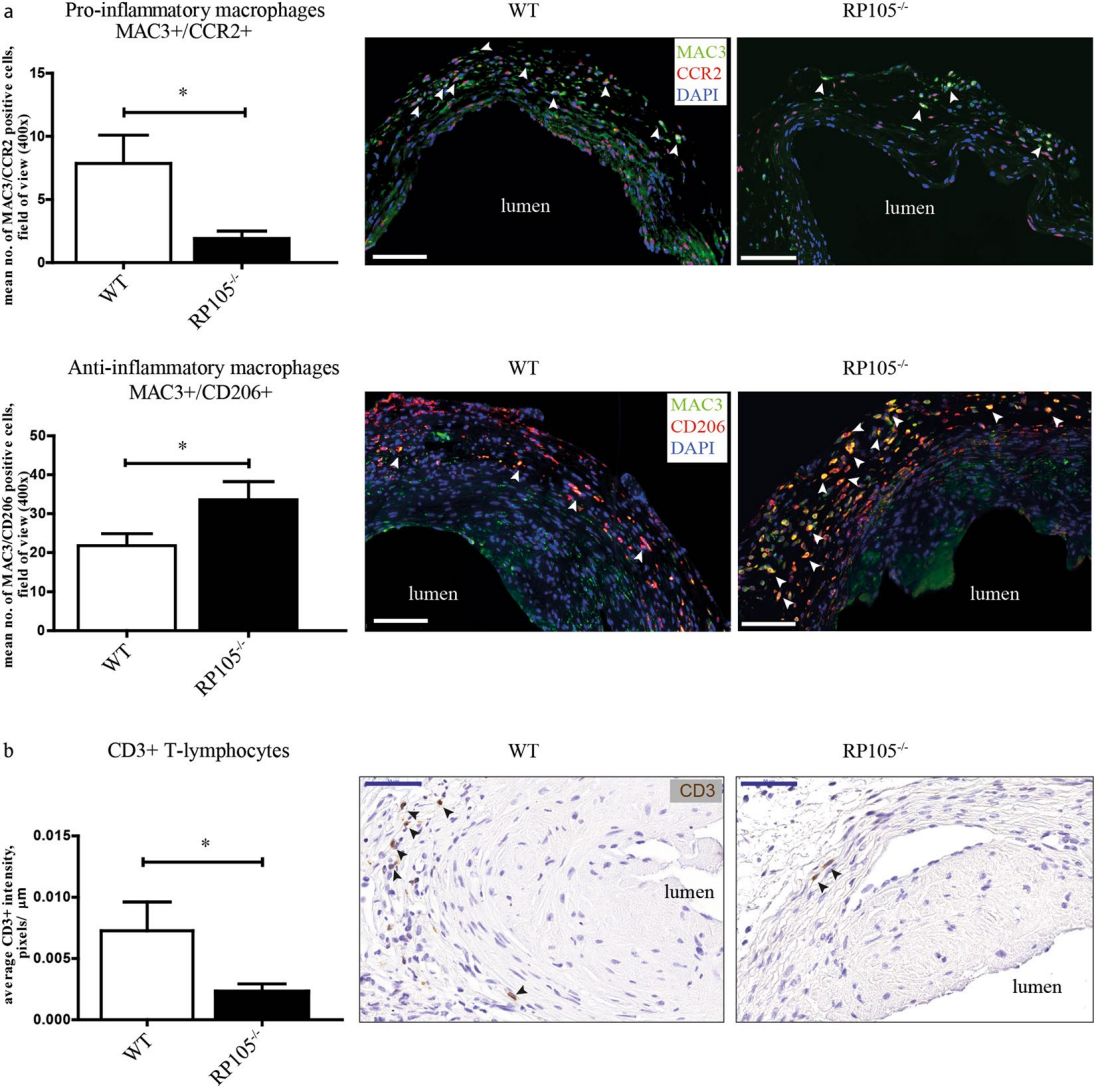
Effects of RP105 deficiency on inflammatory response *in vivo*. Quantification and immunohistochemical staining of (**a**) MAC3^+^/CCR2^+ ^ macrophages and MAC3^+^/CD206^+^ macrophages (white arrows) and (**b**) CD3^+^ T-lymphocytes (black arrows) in AVF lesions 14 days after surgery. Decrease in cell number of pro-inflammatory macrophages and upregulation in anti-inflammatory macrophages upon RP105 deletion was observed. Bar = 100 μm. Number of CD3 T-lymphocytes was reduced in RP105 as compared to WT. Bar = 50 μm; 400x magnification. ^*^P < 0.05; n = 11 per group.

To gain insight into the consequences of differential expression of RP105 on the inflammatory response to injury *in vivo*, we evaluated the inflammatory cell composition of AVFs in RP105^−/−^ and WT mice. Analysis of AVF material 2 weeks after surgery revealed a 76% reduction in MAC3^+^/CCR2^+^ pro-inflammatory macrophages cell number in the venous lesions of RP105^−/−^ mice. Furthermore, the number of infiltrating MAC3^+^/CD206^+^ anti- inflammatory macrophages was increased by 35%, as compared to WT mice (Fig. 6a). The number of CD3^+^ T-lymphocytes in RP105^−/−^ mice was decreased by 70% (Fig. 6b). Interestingly, we observed an enrichment of these inflammatory cells in the adventitial layer of the vessel (Fig. 6a,b). No changes between RP105^−/−^ and WT mice were found in the number of MCP1^+^ cells in the AVF lesions at 2 weeks after surgery (Supplementary Figure 3). Notably, the distribution of the total population of macrophages in RP105^−/−^ 2 weeks after AVF creation was skewed towards a tissue repair, or regenerative state. More than 90% of all MAC3^+^ cells were CD206^+^, a cell surface protein that defines the anti-inflammatory repair associated macrophage phenotype, whereas but 6% of these MAC3^+^ macrophages were found to express CCR2, the pro-inflammatory macrophage marker.

### MMP activity is decreased in AVF lesions of RP105 deficient mice

**Figure 7:**
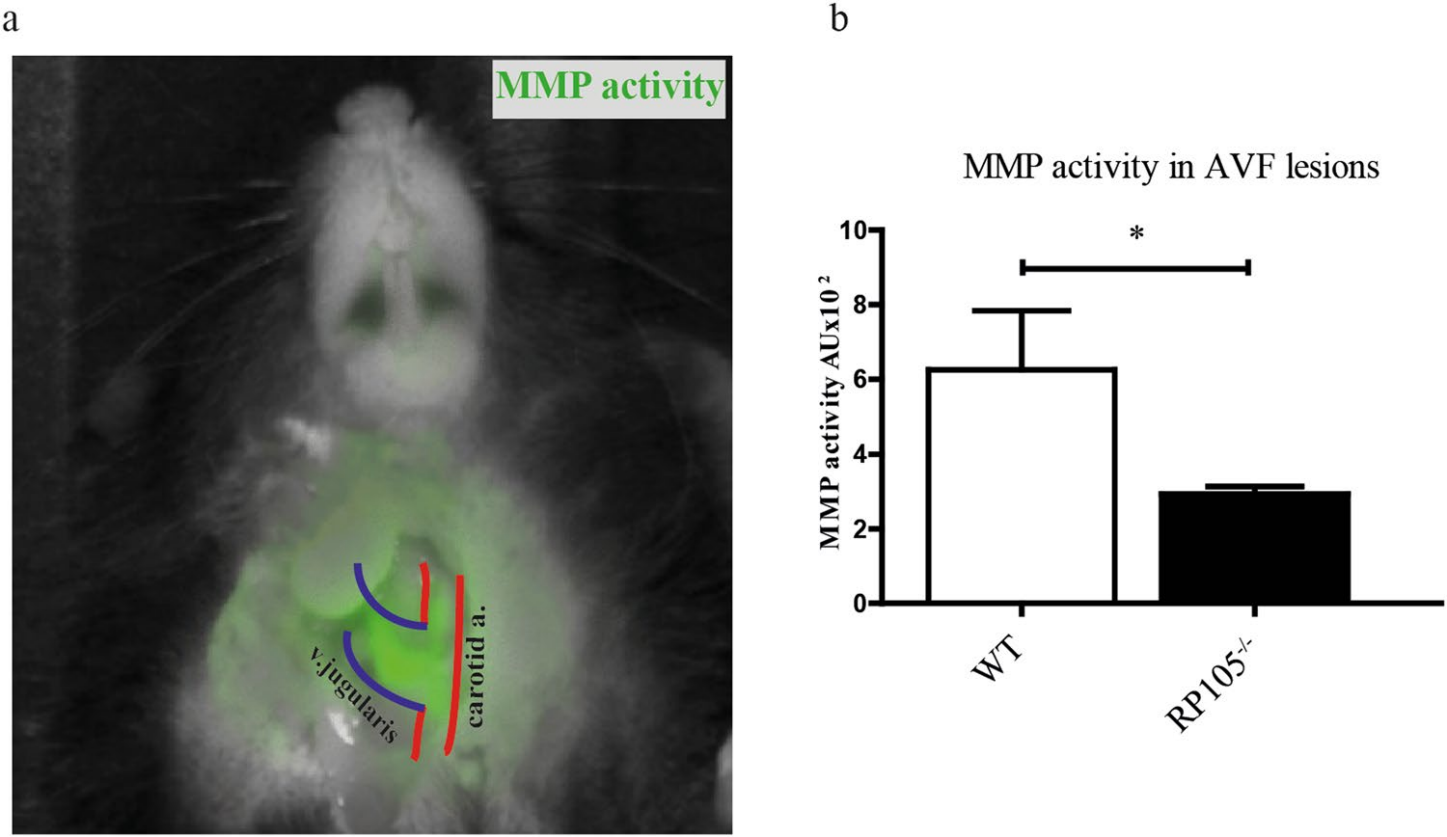
*In vivo* near-infrared biofluorescent imaging and quantitative analysis of MMP activity. (**a**) Quantitative analysis of fluorescent intensity showed decrease in MMP activity in RP105 mice, as compared to WT. (**b**) Visual representation of near-infrared signal from active MMPs. Accumulation of green color can be seen in the anastomotic region 24 h after injection of MMPSenseTM 680 probe. ^*^P < 0.05; n = 4 per group.

Matrix metalloproteinases (MMPs) are known for the role they play in extracellular matrix (ECM) remodeling, such as collagen and elastin. MMP-mediated degradation of the ECM is critically involved in vascular remodeling following AVF placement and during AVF maturation^34^. We assessed MMP activity in the lesions using *in vivo* near-infrared fluorescent imaging. We observed a two-fold reduction (6.3 ± 1.6 WT vs. 2.9 ± 0.2 RP105^−/−^ AU) in fluorescence intensity indicating reduced *in vivo* MMP activity in RP105^−/−^ mice as compared to WT (Fig. 7).

### Macrophage-mediated cytokine production is affected by RP105 expression levels

**Figure 8:**
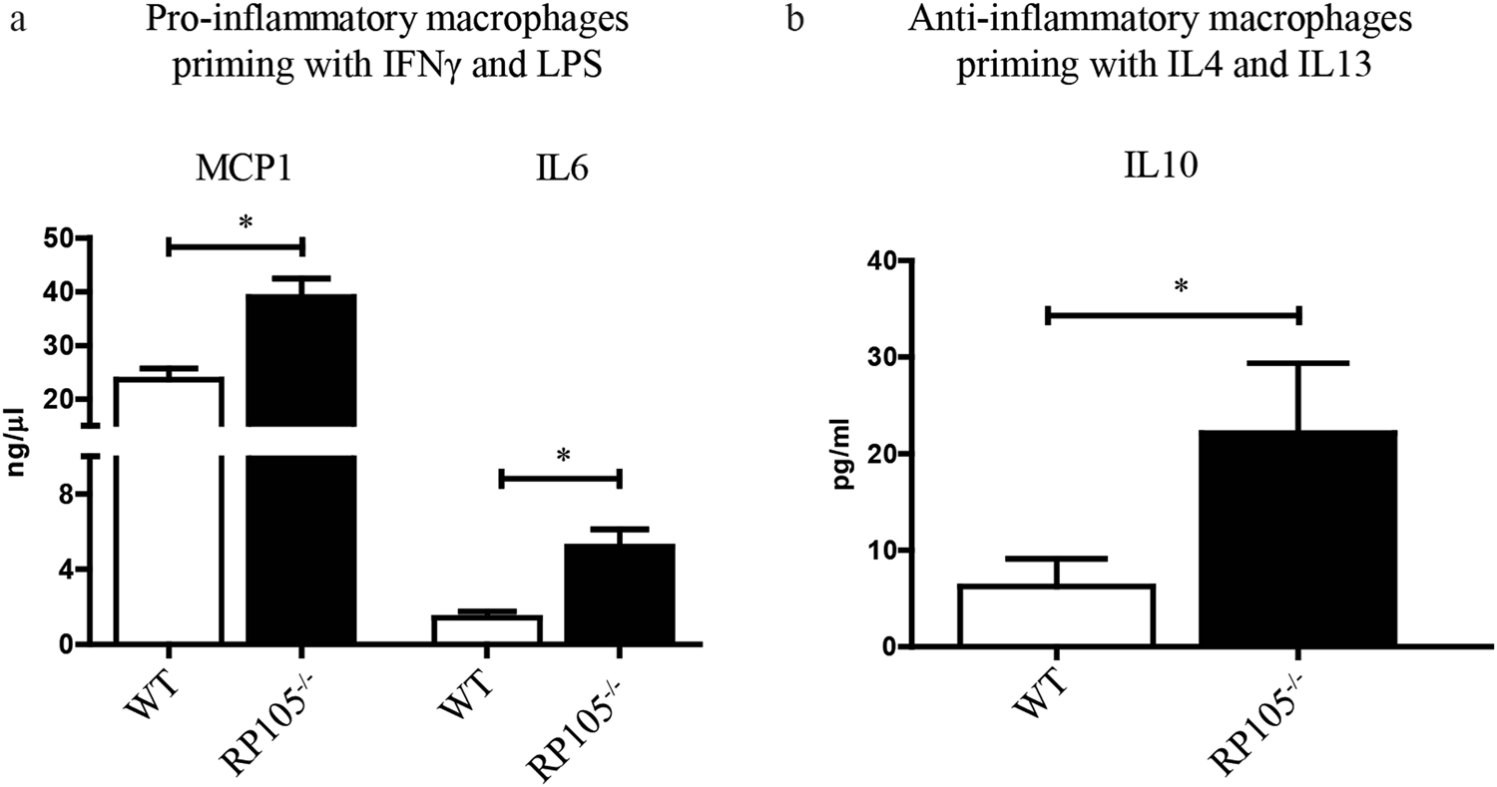
Effect of RP105 deficiency on macrophage function. (**a**) Bone marrow-derived macrophages from RP105 primed towards pro-inflammatory phenotype secrete increased levels of MCP1 and IL6 as compared to WT control mice. (**b**) Anti-inflammatory macrophages from RP105 secrete increased levels of repair associated cytokine IL10. ^*^P < 0.05; n = 3.

Having identified that AVFs in RP105^−/−^ mice are enriched for anti-inflammatory macrophages, we subsequently isolated bone marrow from WT and RP105^−/−^ mice and polarized bone marrow-derived macrophages towards either pro- or anti-inflammatory phenotypes with LPS/IFN-gamma or IL4/IL13 treatment for 24 h, respectively. We observed an augmented inflammatory response by pro-inflammatory macrophages derived from RP105^−/−^ mice as evidenced by a 40% increase in MCP1 secretion and a 73% up regulation in IL6 production, as compared to macrophages obtained from WT mice (Fig. 8a).

Macrophages that were isolated from RP105^−/−^ mice and driven towards the anti-inflammatory phenotype exhibited a 72% increase in anti-inflammatory cytokine IL10 production as compared to WT macrophages (Fig. 8b).

### RP105 is present in the venous wall of human AVF

**Figure 9:**
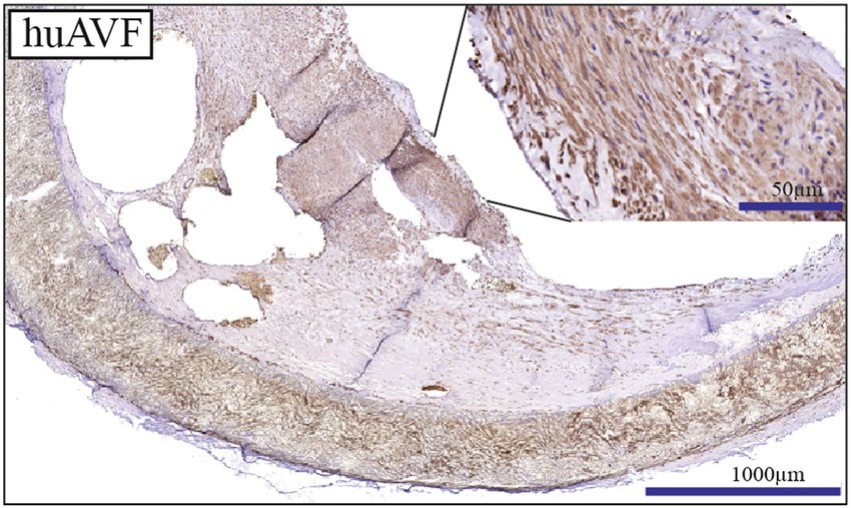
RP105 expression in human AVF. RP105 is highly expressed within the venous neointimal lesions of human AVF n = 4.

Human AVF was obtained in the operating room during AVF correction surgery and processed in the same manner as mouse samples. Immunohistochemical staining of human AVF sections showed an impressive accumulation of RP105 expression within the venous wall. Cells positive for RP105 were mainly located in the neointima (Fig. 9).

## Discussion

In this study, we addressed the specific role of TLR4 homologue RP105 in vascular remodeling, inflammation and VSMCs function in a murine model of AVF failure. The process of AVF maturation is complex and integrates several cellular responses, including the infiltration of inflammatory cells shortly after AVF surgery^5, 35^. In addition, VSMCs play a pivotal role in AVF maturation as they contribute to thickening of the venous vessel wall and the concurrent outward remodeling. Here, we clearly show that RP105 deficiency affects the inflammatory and VSMC-mediated response to injury during the course of AVF maturation, accumulating in an impaired outward remodeling 14 days after the placement of an AVF.

A vital aspect of AVF maturation involves the outward remodeling response, a vessel widening process that is tightly coupled with VSMC proliferation. While VSMC proliferation in IH is generally considered to be detrimental, the process is beneficial for vascular adaptation in AVF, especially in the early phase of AVF maturation. To this end, the reduction in venous outward remodeling in RP105^−/−^ mice, coupled with a reduction in proliferating venous VSMC within AVFs and *ex vivo*, suggests that inhibiting VSMCs proliferation (and migration) could be detrimental for long-term AVF maturation.

A striking observation in our studies was that RP105 diminution differentially affected arterial and venous VSMCs, as evidenced by RP105-specific effects on proliferation and inflammatory cytokine production by venous cells as well as impact on arterial migration. The endogenous expression levels of RP105 in arterial and venous VSMCs support this finding, along with differential expression profiles of associating TLR4-family members (including TLR4 and MD-1). Collectively, these findings suggest that the susceptibility for inflammatory stimuli could potentially differ between arterial and venous VSMCs. Importantly, our studies support the notion that numerous cell sources are involved in venous IH in AVF (including resident venous cells, infiltrating arterial cells, and circulating bone marrow-derived cells^7, 36–39^). Furthermore, our studies illustrate the need for continued investigation of the phenotypic properties and functional characteristics of VSMCs in AVFs, in particular due to the contrasting lineage tracing studies detailing a predominance of arterial VSMCs^10^ versus venous VSMCs^31^ in venous IH following AVF placement.

Increased expression of the pro-inflammatory mediators IL6, TNF and MCP1 are associated with AVF failure^8, 40, 41^, while the reduction of anti-inflammatory molecule heme oxygenase-1 (HO-1) is linked to AVF failure^42–44^. In our study, we demonstrated that the polarization of macrophages isolated from the bone marrow of RP105^−/−^ mice towards the pro- or anti- inflammatory phenotypes appears to remove a regulatory repressor, as both phenotypes displayed an up regulation of signature cytokines being produced. The augmentation of pro-inflammatory cytokine production in RP105^−/−^ macrophages is in keeping with RP105 being an antagonist of pro-inflammatory TLR4 signaling^25, 27, 45^, while the spike in IL10 production is supported by recent reports that low grade inflammation triggers bone marrow-derived macrophages to generate anti-inflammatory cytokines in a TLR4 dependent fashion^46^.

AVF placement in RP105^−/−^ mice yielded decreased MAC3^+^/CCR2^+^ macrophages and CD3^+^ T-lymphocytes. Our previous study performed by Wezel *et al*. on the role of RP105 in atherosclerosis showed the same difference which was linked to decrease in CCR2^+^ monocytes in RP105^−/−^ mice hampering process of monocyte infiltration into the lesions. After additional *in vitro* stimulation with LPS the dose dependent decrease in CCR2 expression on CCR2^+^ monocytes isolated from RP105^−/−^ mice was observed which may point onto increased signaling via TLR4 route^29^. A noteworthy observation two weeks after AVF placement in RP105^−/−^ mice was the attenuation of vessel wall MMP activity. While the type of vascular injury impacts the degree by which MMPs remodel the vascular wall, these factors also play a role in determining which MMPs are activated^46, 47^, and could differ between arterial and venous segments. Castier *et al*. reported that increased MMP-9 activity coincided with increased OR in the arterial segment of the AVF^48^, while Nieves Torres *et al*. demonstrated that MMP inhibition enhanced venous OR in AVF^49^. Our studies contradict this finding, and suggest instead that decreased MMP limits venous OR in maturating AVFs.

During the process of vascular remodeling the initial pro-inflammatory reaction is gradually changing towards resolution of inflammation characterized by accumulation of anti-inflammatory cells^50–53^. The specific dynamics with regard to pro-/anti- inflammatory response in the context of AVF maturation is still unknown. In our murine model, RP105 deficiency caused significant increase in MAC3^+^/CD206^+^ anti-inflammatory macrophages in the venous lesions of AVF, compared to controls. Overall prevalence in anti-inflammatory population (93.7%), compared to 6.3% of pro-inflammatory macrophages at 2 weeks after AVF creation might suggest either that in the current model pro-inflammatory response is completed at earlier time points or that anti-inflammatory macrophages play a dominant role in the tissue response in murine AVF. Thus, despite the increased production of both pro- and anti-inflammatory cytokines by macrophages *in vitro*, the effect of RP105 deletion on anti- inflammatory macrophages was dominant in the venous lesions of murine AVF.

Finally, to our knowledge it is the first study to demonstrate expression of RP105 in human AVF, which is an important observation supporting further research related to RP105 as a potential therapeutic target to improve AVF maturation.

### Study limitations

Current study is performed in mice, which do not precisely mimic the human inflammatory response to injury; however this model remains highly useful for studying the vital pathophysiological aspects of AVF maturation and failure. Another limitation is the absence of uremia, given that a recent *in vivo* study by Kang *et al*. demonstrated that fistula maturation in mice is affected by CKD^44^. Here, the chronic accumulation of waste products and uremic toxins in the blood impacted AVF flow, resulting in increased venous wall thickness and thrombus formation. Also, future studies should include flow measurements, as the rate of blood flow is critical functional parameter of AVF.

In conclusion, our study demonstrates the complex role of RP105 in VSMCs and macrophages in a murine model of AVF. The design and implementation of therapeutic strategies targeting the TLR4/RP105 axis to prevent AVF failure must include cell specific targeting approaches and be temporally controlled.

## Material and Methods

### Animals

#### Murine model of AVF failure

This study was performed in compliance with Dutch government guidelines and the Directive 2010/63/EU of the European Parliament. All animal experiments were approved by the Institutional Committee for Animal Welfare of Leiden University Medical Center. RP105^−/−^ mice (C57BL/6 background) were obtained from the local animal breeding facility, WTC57BL/6 mice were obtained from Charles River. Adult male mice aged 10–11 weeks were used for the experiments. AVF were created in an end-to-side manner between the dorsomedial branch of the external jugular vein and the common carotid artery as previously described^5, 54^ (Supplementary Methods S1). The mice were euthanized at 2 weeks after AVF surgery.

#### *In vivo* near-infrared MMPs assay

*In vivo* MMP activity of endogenous MMP-2, -3, -9, -12 and -13 was assessed by injecting fluorescent imaging agent MMPSense^TM^ 680 from PerkinElmer’s (Waltham, MA, USA) which is activated in the presence of active MMPs^55^. First, AVF was created as described above (n = 4 per group). 14 days later mice were anesthetized under isoflurane and 4 nmol of MMPSense^TM^ 680 probe were injected into the tail vein. 24 hours later, mice were placed under anesthesia, AVF was dissected and mice were scanned using the Optix MX2 optical imaging system. Excitation was performed with a 670-nm pulsing laser, and emission was detected with a 693-nm long-pass filter. Lifetime analysis was used to confirm the specificity of MMP-activated probes. Fluorescence intensities and fluorescence lifetime were expressed in pseudo colors and projected on the bright field grayscale image of the mouse. Quantification of the fluorescent intensity was performed using the Optiview 2.2 software as described previously^56^.

#### Tissue harvesting and processing

14 days after surgery, the mice were anesthetized using isoflurane whereupon the AVF was dissected. After a thoracotomy, the inferior vena cava was transsected followed by a mild pressure perfusion fixation with 4% formalin through an intracardiac perfusion. The tissue was embedded in paraffin and 5 μm-thick sections of the venous outflow tract were made perpendicular to the vein with an interval of 150 μm.

#### Morphometric and histological analysis

Morphometric analysis was performed on Weigert’s elastin stained sections using ImageJ software. Vessel circumference as a parameter displaying the process of outward remodeling was determined by measuring the length of the internal elastic lamina (IEL). The intimal hyperplasia was calculated by subtracting the luminal area from the area within the IEL. Immunohistochemical staining was performed for macrophages (MAC3, 1:200, BD- Pharmingen, SanDiego, USA) in a combination with CCR2 for pro-inflammatory phenotype (1:400, Abcam, Cambridge, UK) or CD206 (1:1000, Abcam) for anti-inflammatory phenotype, T-lymphocytes (CD3, 1:300, Abcam) and VSMCs (αSMA, 1:1000, Dako, Glostrup, Denmark) in a combination with Ki67 (1:200, Abcam) to detect proliferating cells. For the immunohistochemical analysis of the MAC3/CD206, MAC3/CCR2 and αSMA/Ki67 staining, the number of positive cells was counted in 3 random fields of view using a 400x magnification from which the mean was calculated. Quantification of CD3^+^ cells was performed with ImageJ software by calculating % DAB positive area from the total vessel area. All immunohistochemical quantifications were performed on the first 3 venous sections starting from the anastomosis per AVF. Slides were digitized using an automated microscopic scanner (Panoramic digital MIDI, 3DHISTECH, Hungary). Results are expressed as mean ± standard error of the mean.

### Cell culture

#### Vascular smooth muscle cells

Primary arterial and venous vascular smooth muscle cells were isolated from murine carotid artery and vena cava of C57Bl/6 and RP105^−/−^ mice (n = 3 per group) respectively. Connective tissues were removed and vessels cut open. Endothelial monolayer was detached by gentle scraping with sterile surgical forceps. The carotid artery and caval vein were dissected into small pieces and plated onto Petri dish 100 mm or 60 mm diameter coated with 0.1 mg/ml fibronectine. After 14 days of culture with DMEM medium supplemented with 20% FCS, 2 mmol/l l-glutamine, 100 U/ml penicillin and 100 μg/ml streptomycin, cells were trypsinized and re-plated onto 6 or 12 well plates and left for 7 days in culture. Upon enrichment in 80–90% confluence VSMCs were trypsinized and seeded at required density for further functional assays.

### Macrophages

Macrophages were derived from bone marrow by flushing tibia and femur of healthy C57Bl/6 or RP105^−/−^ mice (n = 3 per group) and seeded at a density of 500.000 cells/well in 6- wells plates. Cells were cultured for 7 days in RPMI GlutaMax (Gibco) supplemented with 100 U/ml penicillin/streptavidin, 25% Fetal Calf Serum (FCS) and 20 mg/ml M-CSF (Myltec Biotechnologies) as described previously^6^. On day 7, cells were stimulated either with LPS (100 ng/ml) and IFN-gamma (10 ng/ml) to differentiate them towards pro-inflammatory phenotype or with IL4 (10 ng/ml) and IL13 (10 ng/ml) (all from Preprotech) for anti-inflammatory phenotype. After 24 hours the supernatants were collected for ELISA assays and cells were lysed with Trizol reagent (Invitrogen, Carlsbad, CA, USA) for RNA isolation.

### VSMC proliferation assay

Murine VSMCs, explanted from aortas and veins of control or RP105^−/−^ mice, were subsequently cultured as described above, and proliferation was measured using neutral red cell proliferation and cytotoxicity assay kit from Boster Bio (Pleasanton, CA, USA) according to the manufacturer protocol (Supplementary Methods S2).

### VSMC migration assay

Primary arterial and venous VSMCs from control and RP105^−/−^ mice were grown to confluence and then made quiescent in cultured medium supplemented with 1% FCS for 24 hours. Cells were detached from the surface using Accutase Cell Detachement Solution (Innovative Cell Technologies, Inc., San Diego, CA, USA) and suspended at a concentration of 100.000 cells/ml in culture medium supplemented with 1% FCS. Migration was assayed with a polycarbonate membrane inserts having 8 μm-pores in 24-well chemotaxis chambers using commercial CytoSelect Cell Migration Assay Kit (Cell Biolabs, Inc., San Diego, CA, USA) over 16 hours towards the 20% FCS gradient. All migratory cells were lysed and labeled with fluorescent dye (CyQuant GR). Quantification was performed on a fluorescence plate reader at 480 nm/520 nm.

### ELISA assays

ELISA assays for MCP1, IL6 and IL10 production were performed with cell free supernatant collected from bone marrow-derived macrophages after 24 hours polarization towards pro- or anti-inflammatory phenotype or *ex vivo* cultured arterial and venous VSMCs using commercial available kits following the instructions of the manufacturer (BD Biosciences, San Jose, CA, USA: MCP1- Catalog No. 555260; IL6- Catalog No. 555240, IL10- Catalog No. 555252).

### RT-PCR

Total RNA was extracted from the macrophages and VSMCs using Trizol reagent (Invitrogen) according to the manufacturer’s protocol. RNA was reverse transcribed using a 5-minute 65 °C incubation of 1 µg total RNA with deoxyribonucleotide triphosphates (Invitrogen) and random primers (Invitrogen). c-DNA was synthesized using an M-MLV First-Strand Synthesis system (Invitrogen), and used for quantitative analysis of mouse genes (Supplementary Table 1) with an SYBR Green Master Mix (Applied Biosystems, Foster City, CA, USA). The relative mRNA expression levels were determined by normalization to murine glyceraldehyde 3-phosphate dehydrogenase (GAPDH) using 2[−ΔΔC(T)] method.

### Statistical analysis

Results are expressed as mean ± SEM and considered statistically significant for p < 0.05; t tests and Mann-Whitney tests for parametric and nonparametric data, respectively, were used as appropriate. All *in vitro* experiments were performed in biological n = 3 in experimental triplicates.

## Electronic supplementary material

Supplementary info
