# Supplementary material for: Deficiency of TLR4 homologue RP105 aggravates outward remodeling in a murine model of arteriovenous fistula failure
Source: Sci Rep. 2017 Aug 31;7:10269. doi: 10.1038/s41598-017-10108-4 (PMC5578984; doi:10.1038/s41598-017-10108-4)
Supplement: Supplementary file 1 — Supplementary info [file 41598_2017_10108_MOESM1_ESM.doc]

**Deficiency of TLR4 homologue RP105 aggravates outward remodeling in a murine model of arteriovenous fistula failure.**

Taisiya Bezhaeva, MSc^1,2^, ChunYu Wong, MD1, 3, Margreet R. de Vries, PhD^2,3^, Eric P. van der Veer, PhD^1,2^, Carla M.A. van Alem, MSc^1,2^, Ivo Que, BSc^4^, Reshma A. Lalai, BSc1, 2, Anton Jan van Zonneveld, PhD^1,2^, Joris I. Rotmans, MD PhD1,2 and Paul H.A.Quax, PhD^2,3^

# Supplementary material

**Supplementary Figure 1. MD2 mRNA expression levels.** The relative expression normalized to GAPDH. n=3 per group.


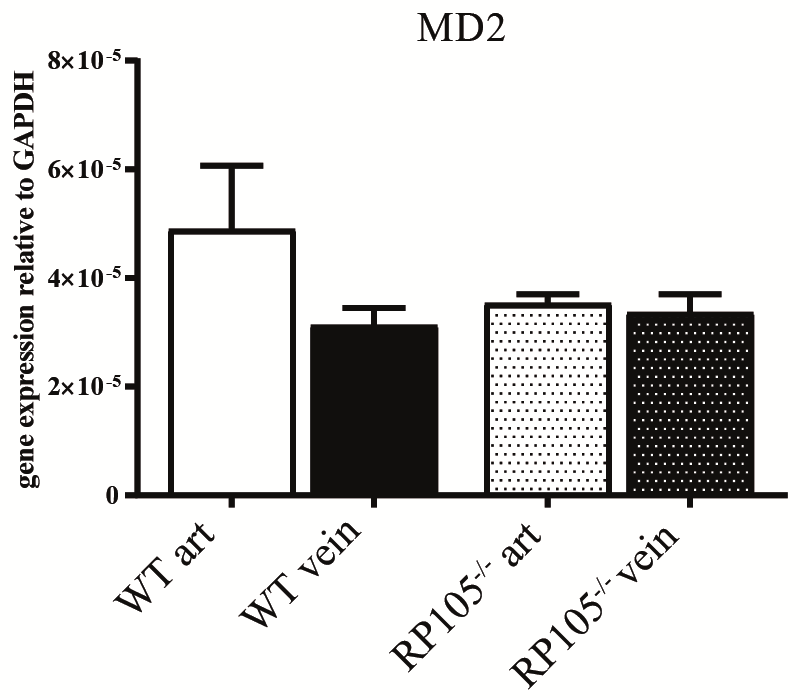


**Supplementary Figure 2. IL10 production by SMCs *in vitro*.** Quantification of anti-inflammatory cytokine IL10 production by in vitro cultured SMCs isolated from WT and RP105-/- . No difference in IL10 levels was detected between RP105-/- and WT mice. n=3 per group.

**
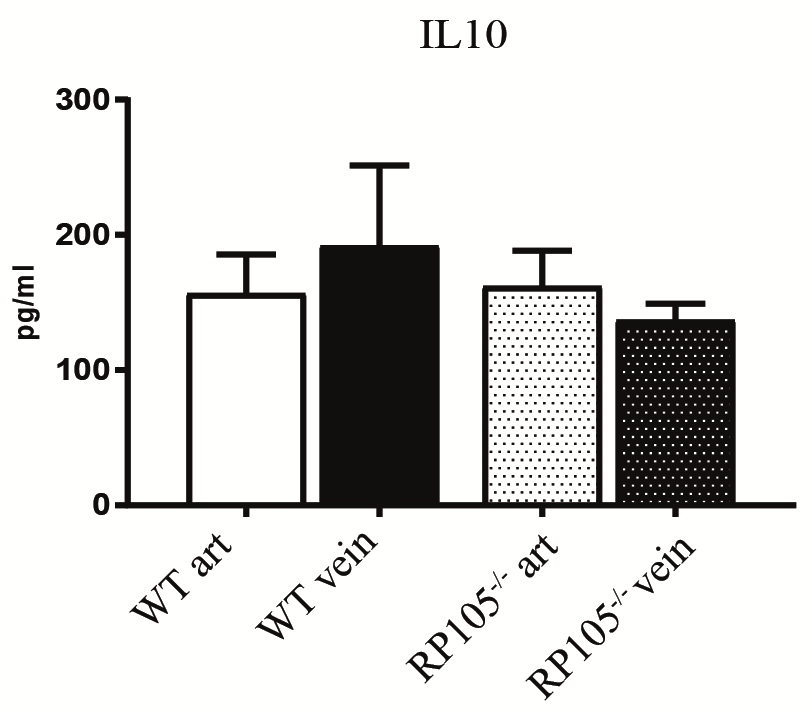
**

**Supplementary Figure 3. Effects of RP105 deficiency on MCP1 expression *in vivo*.** Quantification of MCP+ cells in AVF lesions 14 days after surgery. No difference in MCP1+ cell number was detected between RP105-/- and WT mice. n=11 per group.


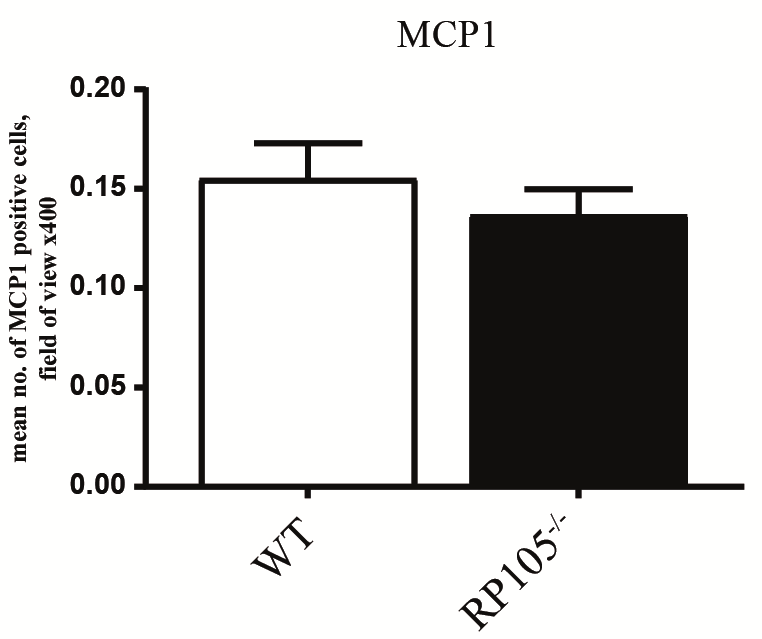


# MATHERIAL AND METHODS

**S1. Surgical procedure**

The animal was anesthetized using isoflurane followed by shaving and disinfection of the skin in the ventral neck area and fixed in a supine position on a heating blanket. The mouse was then injected with buprenorphin (0.1 mg/kg) (MSD, Whitehouse Station, NJ, USA) and 0.5 mL saline. Under a dissecting microscope (Leica, Wetzlar, Germany), an incision in the ventral midline of the neck area was made, followed by a dissection of the right dorsomedial branch of the external jugular vein and ipsilateral common carotid artery after the excision of the sternocleidomastoid muscle using a heat cauterizer. Next, after applying a vascular clamp (S&T, Neuhausen, Switzerland) on the proximal and distal artery an approximate 1 mm incision was made using a microscissor (Fine Science Tools, Heidelberg, Germany) and the lumen was rinsed with a heparin solution (100 IU/ml) (LEO Pharma, Ballerup, LLDenmark). The vein was then clamped proximally and ligated distally, followed by a transection just proximal to the ligation. After rinsing the vein with a heparin solution, an end-to-side anastomosis was created using 10.0 interrupted sutures (BBraun, Melsungen, Germany). Halfway during the suturing procedure, heparin (0.2 IU/gram bodyweight) together with 200 µL of either L-Pred (10 mg/kg bodyweight), Pred (10 mg/kg bodyweight), L-PBS or PBS was injected intravenously. After completion of the anastomosis, the remaining clamps were removed and patency was assessed. The skin was closed with a 6.0 running suture (BBraun, Melsungen, Germany). Following completion of the surgery 0.5 mL of saline was injected subcutaneously and the mice were kept warm until recovery.

# S2. VSMC proliferation assay

5x10^4^ cells per well were seeded in 96 well plates. Next, cells were synchronized overnight in culture medium supplemented with 1% FCS followed by stimulation with 1ng/μl LPS in completed medium supplemented with 20% FCS. Absorbance of neutral red (C15H17IN4) by the cells over a 16 hours period was measured by an ELISA plate reader at A540. Absorbance was compared between the control and knock-out animals with or without stimulation with LPS. All experiments were performed in biological triplicates.

# Table 1

**Primers used for *in vitro* experiments**

| **Gene** | **Forward primer** | **Reversed primer** |
| --- | --- | --- |
| SMA | | CTGACAGAGGCACCACTGAA | | --- | |  | | CATCTCCAGAGTCCAGCACA |
| MYHC | | TGGCTAGCAGCTTGTCAGGAA | | --- | |  | | GCCTTGCGTACTCTATCACTCATG |
| Calponin | | GAAGGCAGGAACATCATTGGA | | --- | |  | | CCTGCTGACTGGCAAACTTG |
| EphB4 | AGTGGCTTCGAGCCATCAAGA | CTCCTGGCTTAGCTTGGGACTTC |
| RP105 | CTTTGAATGCCTCCGTCTTG | GCCCTCTCCACCTTAGACCT |
| TLR4 | TGCCGTTTCTTGTTCTTCC | GAGCTCGGTACTGGCTGTTT |
| MD1 | CTTGGTTATCAGTGGTTCTTGC | AGCGGGATCGAGCCCTC |
| MD2 | CTTACGCTTCGGCAACTCTA | CCTATCCCCTTTGTGAGGAG |
| GAPDH | ACTCCCACTCTTCCACCTTC | CACCACCCTGTTGCTGTAG |
